# Gene Constellation of Influenza A Virus Reassortants with High Growth Phenotype Prepared as Seed Candidates for Vaccine Production

**DOI:** 10.1371/journal.pone.0020823

**Published:** 2011-06-13

**Authors:** Andrew A. Fulvini, Manojkumar Ramanunninair, Jianhua Le, Barbara A. Pokorny, Jennifer Minieri Arroyo, Jeanmarie Silverman, Rene Devis, Doris Bucher

**Affiliations:** Department of Microbiology and Immunology, New York Medical College, Valhalla, New York, United States of America; College of Medicine, Hallym University, Korea, Republic of

## Abstract

**Background:**

Influenza A virus vaccines undergo yearly reformulations due to the antigenic variability of the virus caused by antigenic drift and shift. It is critical to the vaccine manufacturing process to obtain influenza A seed virus that is antigenically identical to circulating wild type (wt) virus and grows to high titers in embryonated chicken eggs. Inactivated influenza A seasonal vaccines are generated by classical reassortment. The classical method takes advantage of the ability of the influenza virus to reassort based on the segmented nature of its genome. *In ovo* co-inoculation of a high growth or yield (hy) donor virus and a low yield wt virus with antibody selection against the donor surface antigens results in progeny viruses that grow to high titers *in ovo* with wt origin hemagglutinin (HA) and neuraminidase (NA) glycoproteins. In this report we determined the parental origin of the remaining six genes encoding the internal proteins that contribute to the hy phenotype *in ovo*.

**Methodology:**

The genetic analysis was conducted using reverse transcription-polymerase chain reaction (RT-PCR) and restriction fragment length polymorphism (RFLP). The characterization was conducted to determine the parental origin of the gene segments (hy donor virus or wt virus), gene segment ratios and constellations. Fold increase in growth of reassortant viruses compared to respective parent wt viruses was determined by hemagglutination assay titers.

**Significance:**

In this study fifty-seven influenza A vaccine candidate reassortants were analyzed for the presence or absence of correlations between specific gene segment ratios, gene constellations and hy reassortant phenotype. We found two gene ratios, 6∶2 and 5∶3, to be the most prevalent among the hy reassortants analyzed, although other gene ratios also conferred hy in certain reassortants.

## Introduction

Due to the genetic instability of influenza viruses, caused by antigenic drift and periodically antigenic shift, the influenza vaccine needs to undergo yearly reformulations. To date the single most practical method of preventing this acute respiratory disease has been the inactivated influenza vaccine. Beginning in the 1940s, the first inactivated vaccines were produced using field isolates, termed wild type (wt) viruses. Wt virus was continuously passaged in embryonated chicken eggs in order to obtain virus with the growth properties needed for vaccine production [1]–[2]. A major problem confronting vaccine production at that time was the low viral titer obtained from wt virus growth *in ovo*.

The influenza A virus is a negative sense, single stranded RNA virus. Its genome is composed of eight individual RNA segments that encode eleven proteins. Segments 4 and 6 encode the two surface glycoproteins, hemagglutinin (HA) and neuraminidase (NA), respectively. The remaining six gene segments encode internal and non-structural viral proteins. The ribonucleoprotein complex is composed of proteins encoded by: segment 1 (PB2), segment 2 (PB1: this segment also encodes the pro-apoptotic protein PB1-F2), segment 3 (PA) and segment 5 (NP). The shortest gene segments, namely 7 and 8, are bicistronic. Segment 7 encodes the matrix protein (M) and an ion channel protein (M2). Segment 8 encodes nonstructural protein 1 (NS1) and nuclear export protein/nonstructural protein 2 (NEP/NS2) [3]. The segmented structure of the genome allows for the exchange of the individual gene segments between influenza viruses, a process defined as reassortment.

In 1969, Edwin D. Kilbourne developed a method, now referred to as the classical method, to prepare seed strains with an *in ovo* high growth phenotype for preparation of influenza vaccines. The classical method takes advantage of the segmented structure of the influenza genome and its ability to reassort to produce high yield (hy) reassortant influenza viruses for vaccine production [4]. A hy reassortant influenza vaccine virus must contain the HA and NA genes of the wt target virus and have the ability to grow to high titers *in ovo*. Hy reassortant vaccine viruses are generated by co-inoculation of two viruses *in ovo*. The two viral parents being the wt virus, which is the current circulating virus that causes disease in a population in a particular year and the hy donor virus that is well adapted for growth in embryonated chicken eggs resulting in high viral titers. Concomitant infection allows for reassortment of genetic segments between the two viruses. Antiserum is used for negative selection against the hy donor surface glycoproteins and in conjunction, adaptation to the host results in positive selection for growth. The reassortant virus that is fittest outgrows other viruses, thereby making this an evolutionary approach. The wt virus provides the HA and NA gene segments, which encode the surface antigens of the reassortant vaccine virus that elicit the immune system to generate protective antibodies. The hy donor virus provides the gene segments necessary to produce an *in ovo* high growth phenotype [4]–[6]. The classical method revolutionized the influenza vaccine manufacturing process.

An alternative approach to preparing seed virus for the influenza vaccine is through reverse genetics. This process is based on incorporating the six internal genes of the hy donor virus and the two genes encoding the surface glycoproteins, HA and NA, from the circulating wt virus into plasmids [7]–[11]. The plasmids are subsequently transfected into cells to rescue the seed virus. The seed virus generated by the reverse genetics method is then inoculated into embryonated chicken eggs for vaccine manufacturing. This system allows for the direct genetic manipulation of the influenza gene segments. The avian influenza virus (H5N1) vaccine has been prepared using reverse genetics technology, as the HA gene segment of H5N1 needed to be modified to reduce its virulence and permit growth of the virus in embryonated chicken eggs for vaccine production [12]–[13].

A/Puerto Rico/8/1934 (PR8) is an H1N1 subtype that is highly adapted to growth in embryonated chicken eggs. For the inactivated influenza A vaccine both the classical method and the reverse genetics technology implement PR8 or its gene segments as their hy donor backbone. PR8 has been used as the hy donor virus since the first hy reassortant virus, X-31b, was used in a commercial influenza virus vaccine [4], [14]–[15]. It has been observed that reassortants that maintain the M gene segment derived from PR8 have a hy phenotype [16]–[17]. Less is known about the contribution of the remaining five internal genes to the *in ovo* hy phenotype. Previous studies have shown that in addition to the M gene, the other internal genes in different combinations also contribute to the *in ovo* hy phenotype [16], [18]–[20].

The present study analyzed the parental origin of each of the eight gene segments for a panel of fifty-seven influenza A hy reassortant viruses that were either vaccine candidates or used in commercial vaccine production. Of the fifty-seven reassortants, 19 are H1N1 subtype and 38 are H3N2 subtype. The study also includes the analysis of vaccine candidates and seed viruses used for the 2009 H1N1pdm vaccine. The analysis was conducted using reverse transcription-polymerase chain reaction (RT-PCR) and restriction fragment length polymorphism (RFLP) [19], [21]–[22]. Characterizing the gene segment ratios and gene constellations that generate hy reassortants in embryonated chicken eggs should assist in selecting the optimal reassortant seed strains needed for vaccine production or in generating seed strains by reverse genetics that will permit optimal growth.

The primary focus of this study was to evaluate the presence or absence of correlations between specific gene segment ratios, gene constellations and the hy reassortant phenotype in order to further improve upon vaccine production strategies. Two gene ratios, i.e. 6∶2 and 5∶3 were the most prevalent found in the fifty-seven hy reassortants analyzed. The wt PB1 gene segment was the most frequent wt gene present in all gene ratios except 6∶2. In contrast the hy donor gene segment that was most prevalent was the M gene segment, which was present in all gene ratios and found in fifty-five out of fifty-seven hy reassortants analyzed.

## Results

### Subtype composition of hy reassortants and hy donor viruses

In this study fifty-seven hy vaccine candidate reassortants generated from thirty-one wt viruses were analyzed. To qualify as a vaccine candidate reassortants must have a titer fold increase greater than or equal to two compared to the respective wt parent. Reassortants that did not fulfill this requirement were not considered vaccine candidate viruses and were not analyzed in this study. Therefore in this study the number of reassortants analyzed from a single wt virus ranged from one to six.

Nineteen reassortants were derived from wt viruses that were H1N1 subtype and thirty-eight were derived from wt viruses that were H3N2 subtype (Table 1). Three hy donors were used to generate the reassortants. PR8, subtype H1N1, HA titer 4096, was used to generate the H3N2 reassortants. In contrast alternative hy donors were used for the H1N1 reassortants, in order to have a clear antigenic difference in the HA and NA subtype between the hy donor and wt viruses, thereby allowing for optimal negative selection. Two exceptions, X-53 and X-53a which are H1N1 reassortants were generated employing PR8 as the hy donor. Two different hy donors, specifically X-31b and NYMC X-157, were used for the generation of the H1N1 reassortants.

**Table 1:** HY Reassortants: Gene Constellations and Fold Increase in HA Titer.

Reassortants	Wild Type Virus	HA Titer* [FI]**	Gene Constellation of HY Reassortants								Gene Ratio
		(hy reassortant HA/wt HA)	PB2	PB1	PA	HA	NP	NA	M	NS	(hy donor∶wt virus)
**H1N1** (hy donor PR8 was used for X-53, X-53a; hy donor X-31b was used for X-127, X-139; hy donor NYMC X-157 was used for the remaining H1N1 reassortants)											
X-53	A/New Jersey/11/1976	512/16 [32]	P	*WT*	P	*WT*	P	*WT*	P	P	5∶3
X53a^a^	A/New Jersey/11/1976	8192/16 [512]	P	*WT*	P	*WT*	P	*WT*	P	P	5∶3
X-127^a^	A/Beijing/262/1995	1024/256 [4]	P	*WT*	P	*WT*	P	*WT*	P	P	5∶3
X-139	A/New Caledonia/20/1999	512/128 [4]	P	*WT*	P	*WT*	*WT*	*WT*	P	P	4∶4
NYMC X-163	A/St. Petersburg/8/2006	2048/32 [64]	P	P	P	*WT*	P	*WT*	P	P	6∶2
NYMC X-163A	A/St. Petersburg/8/2006	2048/32 [64]	P	P	P	*WT*	P	*WT*	P	P	6∶2
NYMC X-163B	A/St. Petersburg/8/2006	1024/32 [32]	P	P	P	*WT*	P	*WT*	P	P	6∶2
NYMC X-173	A/South Dakota/06/2007	4096/128 [32]	P	P	P	*WT*	P	*WT*	P	P	6∶2
NYMC X-173A	A/South Dakota/06/2007	4096/128 [32]	P	P	P	*WT*	P	*WT*	P	P	6∶2
NYMC X-173B	A/South Dakota/06/2007	4096/128 [32]	P	P	P	*WT*	P	*WT*	P	P	6∶2
NYMC X-173C	A/South Dakota/06/2007	4096/128 [32]	P	P	P	*WT*	P	*WT*	P	P	6∶2
NYMC X-177	A/Hong Kong/1870/2008	2048/256 [8]	P	P	P	*WT*	P	*WT*	P	P	6∶2
NYMC X-177A	A/Hong Kong/1870/2008	4096/256 [16]	P	P	P	*WT*	P	*WT*	P	P	6∶2
NYMC X-177B	A/Hong Kong/1870/2008	2048/256 [8]	P	P	P	*WT*	P	*WT*	P	P	6∶2
NYMC X-179	A/California/07/2009	4096/64 [64]	P	*WT*	P	*WT*	P	*WT*	P	P	5∶3
NYMC X-179A^b^	A/California/07/2009	2048/64 [32]	P	*WT*	P	*WT*	P	*WT*	P	P	5∶3
NYMC X-181^b^	A/California/07/2009	4096/64 [64]	P	*WT*	P	*WT*	P	*WT*	P	P	5∶3
NYMC X-181A	A/California/07/2009	4096/64 [64]	P	*WT*	P	*WT*	P	*WT*	P	P	5∶3
NYMC X-181B	A/California/07/2009	2048/64 [32]	P	*WT*	P	*WT*	P	*WT*	P	P	5∶3
**H3N2** (hy donor PR8 virus was used in the reassortment of the H3N2 viruses)											
X-117^a^	A/Beijing/32/1992	4096/512 [8]	P	P	P	*WT*	P	*WT*	P	P	6∶2
X-121^a^	A/Shangdong/9/1993	512/128 [4]	P	P	P	*WT*	P	*WT*	P	P	6∶2
X-123	A/Johannesburg/33/1994	2048/64 [32]	P	P	P	*WT*	P	*WT*	P	P	6∶2
X-137	A/Moscow/10/1999	512/32 [16]	P	P	P	*WT*	P	*WT*	P	P	6∶2
X-141	A/Panama/2007/1999	2048/64 [32]	P	P	P	*WT*	P	*WT*	P	P	6∶2
X-143	A/Ulan Ude/01/2000	2048/256 [8]	P	P	P	*WT*	P	*WT*	P	P	6∶2
X-145	A/California/32/1999	512/32 [16]	P	*WT*	P	*WT*	P	*WT*	*WT*	P	4∶4
NYMC X-147^a^	A/Wyoming/03/2003	1024/16 [64]	P	P	P	*WT*	P	*WT*	P	P	6∶2
NYMC X-149	A/Wyoming/03/2003	256/16 [16]	*WT*	*WT*	*WT*	*WT*	P	*WT*	P	P	3∶5
NYMC X-151	A/Fujian/445/2003	2048/256 [8]	*WT*	P	P	*WT*	*WT*	*WT*	P	*WT*	3∶5
NYMC X-153	A/Texas/40/2003	1024/64 [16]	P	P	P	*WT*	*WT*	*WT*	P	P	5∶3
NYMC X-155	A/Wellington/01/2004	2048/64 [32]	P	P	P	*WT*	P	*WT*	P	P	6∶2
NYMC X-157^a^	A/New York/55/2004	2048/64 [32]	P	P	P	*WT*	P	*WT*	P	P	6∶2
NYMC X-157A	A/New York/55/2004	2048/64 [32]	P	*WT*	P	*WT*	P	*WT*	P	P	5∶3
NYMC X-157B	A/New York/55/2004	1024/64 [16]	P	P	P	*WT*	P	*WT*	P	P	6∶2
NYMC X-157C	A/New York/55/2004	1024/64 [16]	P	P	P	*WT*	P	*WT*	P	P	6∶2
NYMC X-157D	A/New York/55/2004	2048/64 [32]	P	P	P	*WT*	P	*WT*	P	P	6∶2
NYMC X-157E	A/New York/55/2004	128/64 [2]	*WT*	*WT*	*WT*	*WT*	P	*WT*	*WT*	*WT*	1∶7
NYMC X-159	A/Mississippi/05/2004	1024/64 [16]	P	P	P	*WT*	P	*WT*	P	*WT*	5∶3
NYMC X-161^a^	A/Wisconsin/67/2005	512/64 [8]	*WT*	*WT*	*WT*	*WT*	*WT*	*WT*	P	*WT*	1∶7
NYMC X-161B^a^	A/Wisconsin/67/2005	1024/64 [16]	P	P	P	*WT*	P	*WT*	P	P	6∶2
NYMC X-165	A/Nepal/921/2006	2048/128 [16]	P	*WT*	P	*WT*	P	*WT*	P	P	5∶3
NYMC X-167	A/Wisconsin/03/2007	256/16 [16]	P	*WT*	P	*WT*	P	*WT*	P	P	5∶3
NYMC X-169	A/Brisbane/09/2006	2048/16 [128]	P	P	P	*WT*	P	*WT*	P	P	6∶2
NYMC X-171	A/Brisbane/10/2007	1024/128 [8]	*WT*	*WT*	*WT*	*WT*	*WT*	*WT*	P	P	2∶6
NYMC X-171A	A/Brisbane/10/2007	256/128 [2]	P	*WT*	P	*WT*	P	*WT*	P	*WT*	4∶4
NYMC X-171B	A/Brisbane/10/2007	512/128 [4]	P	P	P	*WT*	P	*WT*	P	P	6∶2
NYMC X-171C	A/Brisbane/10/2007	1024/128 [8]	P	P	P	*WT*	*WT*	*WT*	P	P	5∶3
NYMC X-171D	A/Brisbane/10/2007	1024/128 [8]	P	P	P	*WT*	*WT*	*WT*	P	P	5∶3
NYMC X-175A	A/Uruguay/716/2007	512/128 [4]	P	*WT*	P	*WT*	P	*WT*	P	P	5∶3
NYMC X-175C^a^	A/Uruguay/716/2007	2048/128 [16]	P	P	P	*WT*	P	*WT*	P	P	6∶2
NYMC X-183	A/Wisconsin/15/2009	1024/16 [64]	P	P	P	*WT*	P	*WT*	P	P	6∶2
NYMC X-185	A/Guangdong-Luohu/1256/2009	512/64 [8]	P	P	P	*WT*	P	*WT*	P	P	6∶2
NYMC X-187^a^	A/Victoria/210/2009	1024/32 [32]	P	P	P	*WT*	P	*WT*	P	P	6∶2
NYMC X-187A	A/Victoria/210/2009	1024/32 [32]	P	P	P	*WT*	P	*WT*	P	P	6∶2
NYMC X-189	A/Hong Kong/26560/2009	512/64 [8]	P	P	P	*WT*	P	*WT*	P	P	6∶2
NYMC X-191	A/Philippines/219/2009	1024/128 [8]	P	P	P	*WT*	P	*WT*	P	P	6∶2
NYMC X-197	A/Brisbane/11/2010	512/32 [16]	P	*WT*	P	*WT*	P	*WT*	P	P	5∶3

*HA Titer is given as reciprocal of viral dilution at titration end point.

**[FI]: fold increase in HA titer over wt parent virus.

a Used in seasonal influenza vaccine production.

b Used in 2009 H1N1pdm vaccine production.

P: hy donor virus A/PR/8/1934 gene. *WT*: wild type virus gene.

X-31b is an H3N2 hy reassortant resulting from a cross between A/Aichi/2/1968 (H3N2 subtype) with PR8 [4]. It has a 6∶2 gene segment ratio, i.e. 6 genes from PR8 and 2 genes, HA and NA, from A/Aichi/2/1968, and HA titer 4096. X-31b was the first reassortant used in an experimental vaccine in 1971 [14]. Two reassortants namely, X-127 (A/Beijing/262/1995) and X-139 (A/New Caledonia/20/1999) were generated using X-31b as the hy donor (Table 1).

NYMC X-157 (H3N2 subtype) resulted from a cross between A/New York/55/2004 (H3N2 subtype) with PR8. It has a 6∶2 gene segment ratio and HA titer 2048. NYMC X-157 was used as the H3N2 component in the 2005–2006 seasonal influenza vaccine. Fifteen reassortants were generated using NYMC X-157 as the hy donor. These include: NYMC X-163, X-163A, X-163B (wt virus A/St. Petersburg/8/2006), NYMC X-173, X-173A, X-173B, X-173C (wt virus A/South Dakota/06/2007), NYMC X-177, X-177A, X-177B (wt virus A/Hong Kong/1870/2008), NYMC X-179, X-179A (wt virus A/California/07/2009) and NYMC X-181, X-181A, X-181B (wt virus A/California/07/2009) (Table 1).

### Viral genome restriction enzyme patterns

Analysis of restriction sites for the thirty-two wt viruses and hy donor viruses was determined using available sequence data from GenBank. Sequence data for each of the eight different viral gene segments was analyzed. Sequences were not available for all the H1N1 and H3N2 wt viruses; therefore a representative virus sequence for each subtype was used for the analysis. The restriction enzymes chosen for the analysis cut at one or two sites yielding two and three fragments respectively. Differentiation between the hy donor virus (PR8, X-31b or NYMC X-157) and the wt virus gene segments was determined on the basis of distinct band sizes resultant from restriction enzyme digestion of each gene segment.

**Figure 1:**
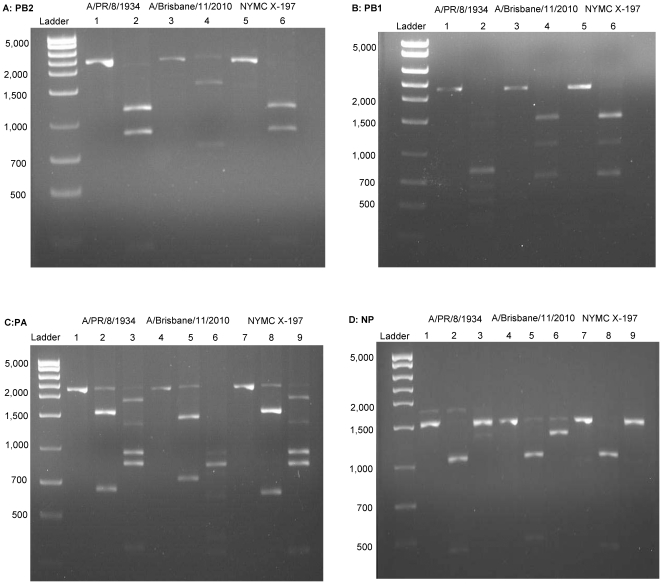
Restriction Fragment Length Polymorphism Analysis of NYMC X-197 Polymerase and Nucleoprotein Gene Segments. NYMC X-197 generated from A/Brisbane/11/2010 (H3N2)×A/PR/8/1934 (H1N1) has a 5∶3 gene ratio, the PB1 gene segment was derived from wt virus; PB2, PA and NP gene segments were derived from A/PR/8/1934. **A.** PB2: lane 1: A/PR/8/1934 undigested; 2: A/PR/8/1934 digested with *Pvu*II; 3: A/Brisbane/11/2010 undigested; 4: A/Brisbane/11/2010 digested with *Pvu*II; 5: NYMC X-197 undigested; 6: NYMC X-197 digested with *Pvu*II. **B.** PB1: lane 1: A/PR/8/1934 undigested; 2: A/PR/8/1934 digested with *Pvu*II; 3: A/Brisbane/11/2010 undigested; 4: A/Brisbane/11/2010 digested with *Pvu*II; 5: NYMC X-197 undigested; 6: NYMC X-197 digested with *Pvu*II. **C.** PA: lane 1: A/PR/8/1934 undigested; 2 and 3: A/PR/8/1934 digested with *Hind*III and *Xmn*I, respectively; 4: A/Brisbane/11/2010 undigested; 5 and 6: A/Brisbane/11/2010 digested with *Hind*III and *Xmn*I, respectively; 7: NYMC X-197 undigested; 8 and 9: NYMC X-197 digested with *Hind*III and *Xmn*I, respectively. **D.** NP: lane 1: A/PR/8/1934 undigested; 2 and 3: A/PR/8/1934 digested with *Hind*III and *Xmn*I, respectively; 4: A/Brisbane/11/2010 undigested; 5 and 6: A/Brisbane/11/2010 digested with *Hind*III and *Xmn*I, respectively; 7: NYMC X-197 undigested; 8 and 9: NYMC X-197 digested with *Hind*III and *Xmn*I, respectively.

**Figure 2:**
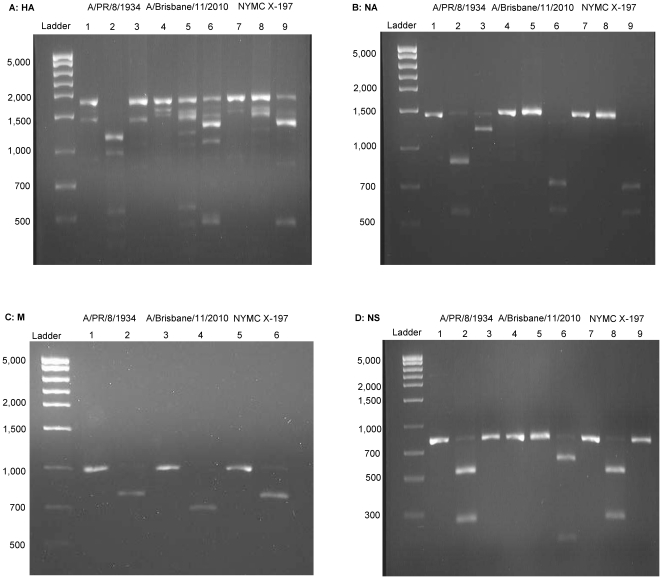
Restriction Fragment Length Polymorphism Analysis of NYMC X-197 Glycoproteins, Matrix and Nonstructural Gene Segments. NYMC X-197 derived the HA and NA gene segments from the wt virus and the M and NS gene segments from A/PR/8/1934. **A.** HA: lane 1: A/PR/8/1934 undigested; 2 and 3: A/PR/8/1934 digested with *Pvu*II and *Hind*III, respectively; 4: A/Brisbane/11/2010 undigested; 5 and 6: A/Brisbane/11/2010 digested with *Pvu*II and *Hind*III, respectively; 7: NYMC X-197 undigested; 8 and 9: NYMC X-197 digested with *Pvu*II and *Hind*III, respectively. **B.** NA: lane 1: A/PR/8/1934 undigested; 2 and 3: A/PR/8/1934, digested with *Bsg*I and *Eco*57I, respectively; 4: A/Brisbane/11/2010 undigested; 5 and 6: A/Brisbane/11/2010 digested with *Bsg*I and *Eco*57I, respectively; 7: NYMC X-197 undigested; 8 and 9: NYMC X-197 digested with *Bsg*I and *Eco*57I, respectively. **C.** M: lane 1: A/PR/8/1934 undigested; 2: A/PR/8/1934 digested with *Bsg*I; 3: A/Brisbane/11/2010 undigested; 4: A/Brisbane/11/2010 digested with *Bsg*I; 5: NYMC X-197 undigested; 6: NYMC X-197 digested with *Bsg*I. **D.** NS: lane 1: A/PR/8/1934 undigested; 2 and 3: A/PR/8/1934 digested with *Sml*I and *Xmn*I, respectively; 4: A/Brisbane/11/2010 undigested; 5 and 6: A/Brisbane/11/2010 digested with *Sml*I and *Xmn*I, respectively; 7: NYMC X-197 undigested; 8 and 9: NYMC X-197 digested with *Sml*I and *Xmn*I, respectively.

A total of six different restriction enzymes were used in the analysis of the eight gene segments (Table 2). One restriction enzyme was used to analyze the gene segments encoding PB2, PB1 and M. Two restriction enzymes were used in the analysis of gene segments encoding PA, HA, NP, NA and NS. Five restriction enzymes, namely *Pvu*II, *Hind*III, *Xmn*I, *Bsg*I and *Sml*I, were used for digestion of multiple gene segments. For example, *Pvu*II was used to digest PB2, PB1 and HA, resulting in a distinct pattern for each gene segment; *Hind*III was used to digest PA, HA and NP, also resulting in a distinct pattern for each gene segment. *Pvu*II showed consistent digestion patterns, albeit at non-canonical cut sites, in the PB1 and PB2 gene segments of the H3N2 subtypes; as well as in the PB1 gene segment of PR8 (data not shown). A representative digestion pattern of the eight gene segments of an H3N2 hy reassortant NYMC X-197 (A/Brisbane/11/2010×PR8) is shown in Figure 1 & 2.

**Table 2:** Restriction Enzymes used for RFLP.

Gene Segment	Enzyme I	Enzyme II
PB2	*Pvu*II	-
PB1	*Pvu*II	-
PA	*Hind*III	*Xmn*I
HA	*Pvu*II	*Hind*III
NP	*Hind*III	*Xmn*I
NA	*Bsg*I	*Eco*57I
M	*Bsg*I	-
	*Sml*I*	-
NS	*Sml*I	*Xmn*I

Gene segments PB2, PB1 and M were digested by a single enzyme.

*SmlI was used to digest 2009 H1N1pdm M gene segment.

### Deviation from the predicted restriction sites within subtypes

Within and among certain subtypes genetic variation, most likely due to point mutations, resulted in different cleavage patterns in contrast to the established cleavage patterns determined for the majority of the wt viruses in a respective subtype. The novel cleavage pattern remained distinct enough to differentiate between the hy donor and the wt virus. Therefore, deviation from predicted restriction sites did not result in ambiguities when determining the origin of a particular gene segment. For example in the PB1 gene segment comparing A/Fujian/445/2003 (NYMC X-151), H3N2 subtype, with other digested H3N2 subtypes two distinct digestion patterns were evident (data not shown). The M and NS gene segments, in distinction to the remaining internal gene segments, showed no variation from the predicted digestion patterns and were found to be conserved among all the H1N1 and H3N2 viral subtypes.

### Gene segment ratios and genetic constellations of hy reassortants

Six distinct gene segment ratios were observed among the fifty-seven hy reassortants analyzed (Table 1 & 3). Gene segment ratios ranged from one gene segment originating from the hy donor and the remaining seven gene segments originating from the wt virus (1∶7, hy donor: wt virus gene ratio), to six gene segments originating from the hy donor and two gene segments originating from the wt virus (6∶2). The 1∶7 gene segment ratio was present in NYMC X-157E and NYMC X-161. The most prevalent gene segment ratio, present in thirty-two reassortants, was six internal gene segments derived from the hy donor and the two surface glycoproteins (HA and NA) originating from the wt virus. The second most prevalent gene segment ratio, present in seventeen reassortants, was five gene segments from the hy donor and three gene segments from the wt virus (5∶3). This gene segment ratio in addition to having the requisite two gene segments for HA and NA originating from the wt virus also had one gene segment encoding an internal protein (i.e. PB1, NP or NS). Three other gene segment ratios were also present in the panel, three reassortants were 4∶4, two reassortants were 3∶5 and one reassortant was 2∶6.

**Table 3:** Gene Constellations Present in Hy Reassortants.

Gene Ratio	Gene Constellations								Frequency
	PB2	PB1	PA	HA	NP	NA	M	NS	
6∶2	P	P	P	*WT*	P	*WT*	P	P	32
5∶3	P	*WT*	P	*WT*	P	*WT*	P	P	13
	P	P	P	*WT*	*WT*	*WT*	P	P	3
	P	P	P	*WT*	P	*WT*	P	*WT*	1
4∶4	P	*WT*	P	*WT*	*WT*	*WT*	P	P	1
	P	*WT*	P	*WT*	P	*WT*	*WT*	P	1
	P	*WT*	P	*WT*	P	*WT*	P	*WT*	1
3∶5	*WT*	*WT*	*WT*	*WT*	P	*WT*	P	P	1
	*WT*	P	P	*WT*	*WT*	*WT*	P	*WT*	1
2∶6	*WT*	*WT*	*WT*	*WT*	*WT*	*WT*	P	P	1
1∶7	*WT*	*WT*	*WT*	*WT*	*WT*	*WT*	P	*WT*	1
	*WT*	*WT*	*WT*	*WT*	P	*WT*	*WT*	*WT*	1

P: hy donor virus A/PR/8/1934 gene. *WT*: wild type virus gene.

Within the six different gene ratio groups twelve separate gene constellations were observed among the fifty-seven hy reassortants (Table 3). The observed distribution of gene constellations (12) was statistically significant (*p*<0.00001) from the expected distribution of 64. The reassortants with a 5∶3 and a 4∶4 gene segment ratio had three distinct gene constellations; while the reassortants that were 3∶5 and 1∶7 had two different gene constellations within their respective gene ratio groups. Since the hy reassortants analyzed were all vaccine candidates the HA and NA must be of wt origin, therefore only one gene constellation was observed among the 6∶2 reassortants. Only a single 2∶6 hy reassortant was found and thus a single gene constellation was determined.

### Hemagglutination titers of hy reassortants in comparison to respective wt virus

Hy reassortants by definition have enhanced growth *in ovo*, as measured by HA titers, when compared with their respective wt virus. HA titers are not a direct measure of viral titers but provide an indication of virus growth or yield. Hy reassortants were defined as having a two fold or greater increase in HA titer compared to the respective wt parent. In this analysis, among the fifty-seven reassortants 2 to 512 fold increases in reassortant HA titers were observed as compared to the wt virus (Table 1). Within the panel of fifty-seven hy reassortants a two fold increase in HA titer was recorded in two reassortants, NYMC X-157E (A/New York/55/2004, 1∶7) and NYMC X-171A (A/Brisbane/10/2007, 4∶4). In contrast, X-53a (A/New Jersey/11/1976, 5∶3) had a 512 fold increase in HA titer.

Fold increases in HA titer varied within reassortant groups that had the same wt virus parent. In three reassortant groups, there was a direct correlation between the greater number of gene segments originating from the hy donor and the higher HA titer. The reassortants generated for the wt virus A/Wisconsin/67/2005, NYMC X-161 (1∶7) and X-161B (6∶2) had an 8 fold and 16 fold increase in HA titer, respectively. Likewise the two reassortants NYMC X-147 (6∶2) and X-149 (3∶5) generated for wt virus A/Wyoming/03/2003 had a 64 fold and 16 fold increase in HA titer respectively. Similarly NYMC X-175A (5∶3) and X-175C (6∶2) both generated from the wt virus A/Uruguay/716/2007 showed an increase in HA titer of 4 fold and 16 fold, respectively. In contrast the NYMC X-171 group had five reassortants that were generated from A/Brisbane/10/2007: X-171 (2∶6), X-171A (4∶4), X-171B (6∶2), X-171C (5∶3) and X-171D (5∶3). For this group HA titer fold increases ranged from 2 to 8 fold, with the highest fold increases found in the 5∶3 and 2∶6 gene ratios.

Four reassortant groups had the same gene ratio and gene constellation and differed in HA titers within the respective group. Both X-53 and X-53a generated from A/New Jersey/11/1976 had a 5∶3 gene ratio, but X-53 had a 32 fold increase while X-53a had a 512 fold increase in HA titer. Three reassortants were generated from wt virus A/St. Petersburg/8/2006, specifically NYMC X-163 (6∶2), X-163A (6∶2) and X-163B (6∶2). X-163 and X-163A had a 64 fold increase in HA titer; in contrast X-163B had a 32 fold increase in HA titer. Likewise, two of the three 6∶2 reassortants generated from A/Hong Kong/1870/2008: NYMC X-177 and X-177B had an 8 fold increase whereas, X-177A had a 16 fold increase in HA titer. Similarly three of the five hy reassortants, all 5∶3, developed from A/California/07/2009 for the 2009 H1N1pdm vaccine, NYMC X-179, NYMC X-181 and X-181A had a 64 fold increase in HA titer whereas NYMC X-179A and X-181B had a 32 fold increase in HA titer. In contrast two reassortant groups had the same gene ratio and gene constellation and the same HA titer fold increase within the respective group. Four H1N1 reassortants produced for A/South Dakota/06/2007: NYMC X-173, X-173A, X-173B and X-173C, all had 6∶2 gene ratios that resulted in a 32 fold increase in HA titer. Likewise NYMC X-187 and X-187A, generated from A/Victoria/210/2009, had 6∶2 gene ratios and a 32 fold increase in HA titer.

## Discussion

Presently inactivated hy reassortants generated in embryonated chicken eggs are used to produce 400 million yearly doses of influenza vaccine [23]. Hy reassortants allow manufacturers to produce the necessary amounts of virus for the seasonal vaccines in a shorter time frame with smaller quantities of embryonated chicken eggs. Although in seasonal epidemics we generally have the infrastructure to provide influenza vaccination to the most at risk groups as well as the general population; production limitations were obvious when manufacturers were confronted with the need to produce the 2009–2010 seasonal vaccine as well as the 2009 H1N1 pandemic vaccine. The current composition of the influenza vaccine is trivalent, with two influenza A components, H1N1 and H3N2, and one influenza B component (Victoria or Yamagata lineage) [24].

A crucial aspect to the vaccine manufacturing process is to obtain influenza A seed virus that has the ability to grow well in embryonated chicken eggs. Currently there are two methods for generating hy influenza A seed strains for vaccine manufacturing, the classical method and reverse genetics. The classical method, which does not use a fixed gene ratio, strictly uses embryonated chicken eggs throughout the process. To date, this is the current method used for the generation of all of the seasonal inactivated influenza A vaccine prepared *in ovo*. Reverse genetics in contrast employs a fixed 6∶2 gene ratio and must rescue its influenza seed virus from cell culture before it can be propagated in embryonated chicken eggs. When growth was evaluated in a direct comparison with the hy reassortants made using the classical method, the reverse genetics reassortants frequently did not show enhanced growth [25]. Although reverse genetics is a powerful tool for the direct manipulation of the gene segments, the classical method has three advantages. First this method allows for natural reassortment of two viruses to take place within the host, thus the reassortant seed virus is highly adapted to the egg host from the earliest stage. Secondly this method does not employ a fixed gene ratio and allows for the selection of the gene ratio and gene constellation that will result in the highest growth phenotype. Lastly the wt target virus and the hy donor A/PR/8/1934 virus are not cloned; therefore variants of all genes including the hemagglutinin and neuraminidase are selected within the egg host to produce the reassortant with the highest growth.

Hy reassortants are the progeny of two distinct influenza viral subtypes. The genome of each parent virus is comprised of eight gene segments and therefore the possible combinations that could result in their progeny are 2^8^ or 256 combinations. However in order to immunize a susceptible population, the two surface glycoproteins must derive their gene segments from the wt virus. Therefore six genes remain available for reassortment, resulting in 2^6^ or 64 possible combinations. In addition to the gene segment combinations resulting from reassortment, pre-existing variants and virus mutations selected during adaptation to the egg host also occur. The importance of these variants in selection for adaptation for high yield is well illustrated by X-53 and X-53a (A/New Jersey/11/1976×PR8), both were 5∶3 reassortants prepared for the swine flu vaccine in 1976. X-53 and X-53a were not antigenically distinguishable, but a single amino acid change in the HA gene increased the yield of X-53a by 16-fold in comparison to X-53 [26]–[28]. With 64 different possible combinations due to reassortment, in addition to variants generated by point mutations, it is important to improve our understanding of which gene segment ratios and gene constellations produce the most efficient hy reassortants.

Baez *et al.* (1980) stated that the genes from the hy donor virus may function in an additive manner and therefore the highest yielding reassortants should contain the majority of the internal genes from the hy donor virus. Generally this held true in our analysis, but there were exceptions. The additive phenomena can be clearly seen in the NYMC X-161 reassortant family, in the NYMC X-175 reassortant family and in the two reassortants NYMC X-147 and X-149. In all three of these reassortant families the greater number of gene segments originating from the hy donor the greater the fold increase in HA titer found for the reassortant. In contrast the NYMC X-171s were an exception to this pattern. Of the five reassortants in this family a greater fold increase was seen in the reassortants that had 5∶3 and 2∶6 gene ratios; in contrast, a lower fold increase was seen in the reassortant with a 6∶2 gene ratio.

Certain hy reassortants generated from the same wt virus and with the same 6∶2 gene ratio differed in their fold increase in HA titer in comparison with their respective low-yield wt virus parent. Two reassortant families, NYMC X-163s and NYMC X-177s, exhibited this characteristic. The difference in HA titer in the hy reassortants generated from a common wt virus and hy donor with the same 6∶2 gene ratio is most likely due to point mutations in one or more of the gene segments producing reassortants with different growth characteristics in embryonated chicken eggs resulting in an increase or decrease of HA titer.

**Figure 3:**
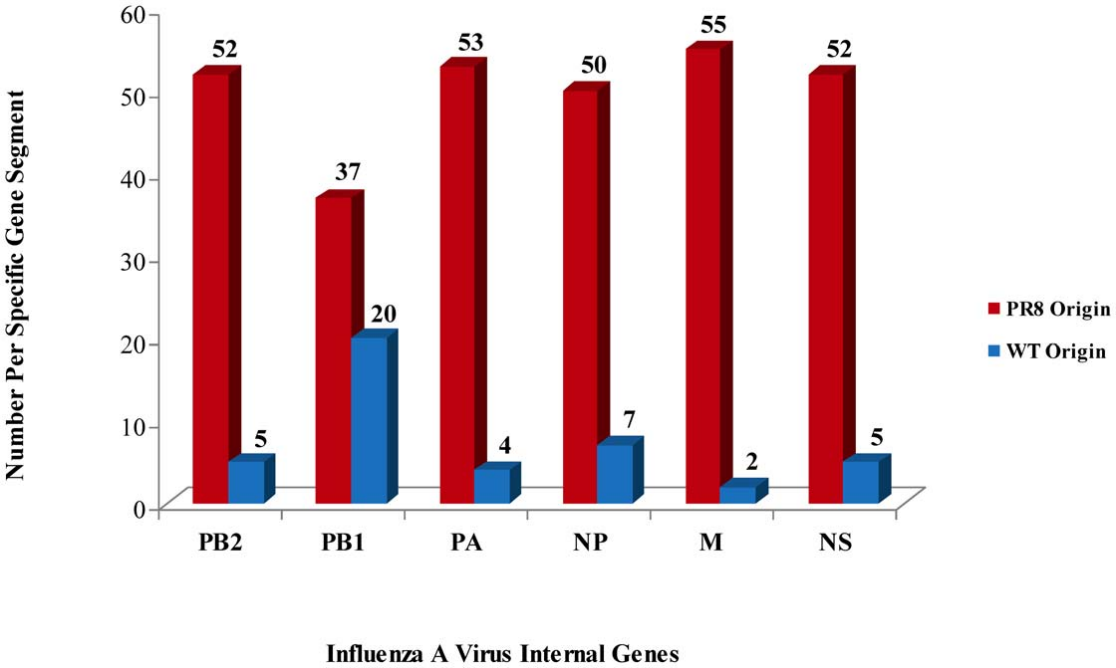
Frequency of Origin of Internal Genes in High Yield Reassortants.

The PB1 gene segment was the most frequent internal wt gene segment present in our panel of hy reassortants (Figure 3). Our analysis showed that in several hy reassortants from both the H1N1 (e.g. NYMC X-179, X-181) and H3N2 (e.g. NYMC X157A, X-165) subtypes the wt origin of the PB1 gene segment produced a high growth phenotype. Enhanced growth of reassortant virus containing wt PB1 was also seen for H5N2 avian viruses. In the analysis by Rudneva *et al.* (2007) the reassortant BC-R22-DP (5∶3) produced from an H5N2×PR8, the PB1 gene was of wt virus origin; BC-R22-DP had higher viral titers as compared to a 6∶2 reassortant generated from the same wt virus. It has also been shown that for 1918 H1N1 wt PB1, in a 1∶7 gene combination made with reverse genetics implementing A/Texas/36/1991, was important for optimal replication, as well as maximal virulence [29].

The reassortants produced from A/California/07/2009 for the 2009 H1N1pdm vaccine all had 5∶3 gene ratios with PB1, HA and NA genes coming from the wt virus. NYMC X-179, X-181 and X-181A had the highest fold increase in this reassortant group. While the fundamental characteristic of hy reassortants is the high growth capacity in comparison to the respective wt virus, they must be antigenically indistinguishable from the respective wt virus in order to be considered a vaccine virus. NYMC X-179 is an example of a vaccine candidate reassortant virus with high yield that upon further analysis was found to have a mutation in the HA resulting in altered antigenicity compared to wt; therefore it was rejected as a vaccine virus [25]. Although the HA titer fold increase was lower for NYMC X-179A in comparison with X-179, it was antigenically indistinguishable from wt virus thereby meeting the requirements to be used as a vaccine virus. In contrast to NYMC X-179, NYMC X-181 and X-181A were antigenically indistinguishable from wt [25]. An additional example of selection for the wt PB1 gene segment in an H1N1 subtype was X-127 developed from A/Beijing/262/1995 and X-31b. X-127 had a 4 fold increase in HA titer in comparison with the respective wt virus.

Within the H3N2 subtype three reassortants, NYMC X-165 generated from A/Nepal/921/2006, NYMC X-167 generated from A/Wisconsin/03/2007 and NYMC X-197 generated from A/Brisbane/11/2010 had a 5∶3 gene ratio that included the respective wt PB1 gene segment and all three had a 16 fold HA titer increase in comparison to wt. Interestingly, within a reassortant family PB1 originating from wt confers the same hy phenotype as PB1 originating from the hy donor PR8. Six reassortants were produced from A/New York/55/2004, NYMC X-157 to X157E. X-157A (5∶3) obtained wt PB1 with the five remaining internal genes from PR8 and has the same titer as X-157 and X-157D, which have 6∶2 gene ratios. In contrast in the NYMC X-175 hy reassortant family this did not hold true. X-175A had a 5∶3 gene ratio, with PB1 originating from the wt virus and a 4 fold increase in HA titer, while X-175C (6∶2) had a 16 fold increase, suggesting that in the X-175s, PB1 originating from PR8 may confer higher titers.

Our study suggests that for some reassortants, a complete polymerase complex comprised of PB2, PB1 and PA from one viral source may provide a growth advantage over a complete polymerase complex from another viral source. The reassortants NYMC X-147 and X-149 were both generated from the same wt virus A/Wyoming/03/2003, but have the polymerase complex composed of the three segments from two different donors, with the remaining genetic background the same. X-147 (6∶2) has the complete polymerase complex from the hy donor PR8; X-149 (3∶5) in contrast has the complete polymerase complex of wt virus origin. The two hy reassortants had very different fold increases in HA titer: X-147 had a 64 fold increase in HA titer whereas X-149 had a 16 fold increase in HA titer. Although X-149 retained all of the polymerase complex subunits from the wt virus, and therefore presumably maintained the functional cooperation between the three subunits, it did not confer the same growth characteristics found when a complete polymerase complex was of PR8 origin. It should be noted that the NP protein is a fundamental component of the ribonucleoprotein complex thus the origin of the NP gene segment may also influence the efficiency of the polymerase complex. NYMC X-149 derived the NP gene from PR8, which may not have interacted with the wt polymerase complex in the most efficient manner in this reassortant.

Among the reassortants with a 5∶3 gene ratio, the second most common gene constellation had the NP gene segment derived from the wt parent (Table 3). The NP gene segment was also the second most common internal wt gene segment present among the fifty-seven reassortants analyzed (Figure 3). Offringa *et al.* (2000) analyzed five hy H3N2 reassortants, two of which, Resvir-9 and X-125 had the same wt virus parent (A/Nanchang/933/1995). The Resvir-9 and X-125 reassortants were produced in different laboratories and both had 5∶3 gene constellations, deriving the NP gene from the wt virus. Our study also found reassortants that had high HA titers while retaining the wt NP gene. Five reassortants were produced for A/Brisbane/10/2007, NYMC X-171 to X-171D. Among the five reassortants, two with the highest HA titers, X-171C (5∶3) and X-171D (5∶3) had the NP gene segment originating from wt virus; in contrast X-171B (6∶2) had a 2 fold lower HA titer. This suggests that the NP gene segment from wt virus contributes to the hy trait for the X-171 reassortant group. The wt virus NP gene segment may also be responsible for contributing the hy phenotype to NYMC X-153 (5∶3), which had an 16 fold increase in HA titer over its' wt parent.

Oxford *et al.* (1978) found the combination of the PR8 PB1 and NP genes contributed hy characteristics to H3N2 reassortants, although they could not determine the origin of the M gene. In our analysis NYMC X-157E (1∶7) acquired only the NP gene from PR8 giving it a 2 fold increase in HA titer over the wt virus; although within the X-157 family the reassortants that acquired the majority of the genes from PR8 had the highest HA titers. In contrast NYMC X-139 (4∶4) an H1N1 reassortant derived the PB1 and NP gene segment from wt virus and had a 4 fold increase in HA titer.

Of the fifty-seven reassortants analyzed, fifty-five reassortants obtained the M gene from PR8 (Table 1). It is well documented in the literature that the M gene segment from a hy donor is needed in order to generate a hy reassortant [16]–[17], [30]. This could be attributable to the multiple functions of this protein. The M1 protein, located on the interior side of the viral envelope, is the most abundant protein in the virion and affects virion morphology. Filamentous viruses have a lower replicative titer in contrast to the higher replication titers seen in viruses with spherical morphology [5]. It has been shown that if a filamentous wt virus is reassorted with PR8 resulting in progeny that have the M gene segment derived from PR8, the reassortants will have spherical morphology [31], perhaps granting a high growth phenotype to the reassortants.

M1 also functions in the transport of the RNP complexes from the nucleus to the cytoplasm. This is a critical step in viral particle assembly. Liu *et al.* (2002) analyzed the binding affinity between M1 and the RNP complex of Resvir-9 (A/Nanchang/933/1995×PR8, 5∶3) in comparison to PR8 and to its' respective wt virus. Their study found that the binding affinity between the RNP complex and M1 of Resvir-9 was similar to that of PR8; a lower binding affinity between the RNP complex and M1 was found for wt A/Nanchang/933/1995. Strong M1/RNP binding may facilitate transport of the RNPs from the nucleus with resultant viral particle assembly and maturation resulting in viral release from infected cells [31]. NYMC X-161 (1∶7), which had only the M gene originating from PR8, had an 8 fold increase in HA titer compared to wt. In another study Klimov *et al.* (1983) analyzed ten hy reassortants that had acquired only the M gene segment from a hy donor and found a strong correlation between the incorporation of the hy donor M gene segment and the hy phenotype.

It was determined that two reassortants in this analysis, NYMC X-145 and NYMC X-157E, derived the M gene from wt rather than from PR8. X-157E had six other wt gene segments in addition to wt M and had the lowest titer in the X-157 family. The second reassortant, X-145 in addition to the wt M gene also obtained the PB1 gene from wt virus, with the remaining internal genes derived from PR8. In this particular case although the M gene originated from the wt virus, a hy reassortant was generated with a 16 fold increase in HA titer. X-145 is the rare exception to the well documented fact that hy reassortants normally obtain the M gene segment from PR8.

The 6∶2 gene ratio with only the HA and NA genes originating from the wt virus is the most prevalent combination among hy reassortants, but it is not the only gene ratio that results in high titers. A fixed gene ratio may not be the best criterion for all influenza vaccine candidates. Rudneva *et al.* (2007) has shown that H5 reassortants with a 5∶3 gene ratio grow to higher titers than H5 reassortants that are 6∶2. In our study, 5∶3 gene ratios also conferred titers as high as 6∶2 gene ratios, and in certain reassortants even higher titers. We also found a single 2∶6 gene ratio and multiple unique gene constellations (Table 3). The optimal gene combinations may facilitate replication through viral RNA and protein interaction with cellular components as well as interaction of viral RNA and protein or protein-protein interactions within the virus. These multi-factorial contributions result in selection of a high replication competent reassortant in embryonated chicken eggs in comparison to the respective low yield wt viruses. Preparation of hy reassortants by classical reassortment allows selection of reassortant viruses *in ovo* with the optimal gene ratio and gene constellation that grant the highest growth. This study demonstrates that the gene ratio and gene constellation yielding a reassortant with hy phenotype is dependent on the individual wt virus and its interaction with the hy donor.

## Materials and Methods

### Viruses

Viruses designated with an X, i.e. X-53 through X-145, have been obtained from the laboratory of Dr. Edwin D. Kilbourne. Viruses designated NYMC X, i.e. NYMC X-147 through NYMC X-197, are from the laboratory of Dr. Doris Bucher. The viral panel used for this study was composed of fifty-seven hy reassortants and their respective wt and hy donor viruses (Table 1). Reassortants were generated according to established methods [4]–[5]. Hy donor virus and a wt virus were co-inoculated *in ovo*. After 42–48 hour incubation at 35°C the allantoic fluid was harvested. The mixed reassortant progeny were subjected to negative selection by the addition of antiserum or purified antibodies prepared from sera of rabbits (PRF&L Inc., Canadensis, PA) immunized with hy donor virus or hy donor virus surface glycoproteins; this was followed by *in ovo* incubation for 42–48 hour at 35°C. The negative selection step was repeated two additional times. The resultant reassortant viruses were harvested and amplified by further incubation cycles without antisera/antibodies. The allantoic fluids were harvested and the reassortant viruses cloned by limiting dilution.

### Hemagglutination Assay

Relative viral titers were determined using a standard hemagglutination assay (HA). 50 µl PBS was added to each well of a V-shaped 96-well microtiter plate. 50 µl of harvested allantoic fluid was added to the first well in column one, serial dilutions were made by transferring 50 µl from the first well of column one to the successive columns, the final 50 µl were discarded. 50 µl of 0.5% chicken red blood cell suspension (PRF&L Inc., Canadensis, PA) was added to each well on the plate. Using a microtiter plate shaker, the contents of the plate were agitated and incubated at room temperature for 30 minutes [32].

### RNA Preparation

RNA was isolated from 280 µl of allantoic fluid using QIAmp® Viral RNA Mini Kit (Qiagen Inc., Valencia, CA) per manufacturer's recommendations. The RNA samples were stored at −20°C until further use.

### Reverse transcription and PCR amplification

Primers were designed in order to include the 3′ and 5′ conserved regions in addition to 8–11 extra nucleotides that were segment specific. Primers were synthesized by Integrated DNA Technologies Inc. (Coralville, IA). Forward (F) and reverse (R) primers used for PCR amplification of gene segments are shown in Table 4. RT-PCR was performed using Takara One Step RNA PCR Kit (Takara Bio Inc., Otsu, Shiga, Japan) per manufacturer's recommendations. Briefly 2 µg of vRNA was added to the following mixture: 10× One Step RNA PCR Buffer, 5mM MgCl_2_, 1mM dNTP, 0.8U/µl RNase Inhibitor, 0.1U/µl AMV RTase XL, 0.1U/µl AMV-Optimized *Taq*, 0.4 µM each of forward and reverse primers, and RNase free H_2_O up to a total volume of 50 µl. RT-PCR parameters used were as follows: 55°C for 30 minutes, 94°C for 2 minutes, followed by 35 cycles of 94°C for 30 seconds, 55°C for 1 minute (HA, NP, NA, M and NS gene segments) or 61°C for 1 minute (PB2, PB1 and PA gene segments), 68°C for 2 minutes and a final extension at 72°C for 10 minutes. The reactions were performed on an Eppendorf Mastercycler® (Eppendorf AG, Hamburg, Germany). The amplified RT-PCR products were visualized on a 2% agarose-TAE/EtBr gel.

**Table 4:** Oligonucleotide Primers used in RT-PCR.

Gene	Primer Sequence	Primer Length (nt)	Annealing Temp. (°C)	Size of Amplicon (∼nt)
PB2	F: AGCGAAAGCAGGTCAATTATATT	23	61	2341
	R: AGTAGAAACAAGGTCGTTTTTAA	23		
PB1	F: AGCGAAAGCAGGCAAACCATTTG	23	61	2341
	R: AGTAGAAACAAGGCATTTTTTCA	23		
PA	F: AGCGAAAGCAGGTACTGATC	20	61	2233
	R: AGTAGAAACAAGGTACTTTTTTG	23		
HA	F: GTTCAGAAAAAGCAGGGG	18	55	1778
	R: AGTAGAAACAAGGGTGTTTT	20		
NP	F: AGCAAAAGACAGGGTAGATAATC	23	55	1565
	R: AGTAGAAACAAGGGTATTTTTC	22		
NA	F: AGCGAAAGCAGGAGTTTAAAAT	22	55	1413
	R: AGTAGAAACAAGGAGTTTTTTG	22		
M	F: AGCGAAAGCAGGTAGATATTGA	22	55	1027
	R: AGTAGAAACAAGGTAGTTTTTT	22		
NS	F: AGCAAAAGCAGGGTGACAAAAA	22	55	890
	R: AGTAGAAACAAGGGTGTTTTTT	22		

F: forward primers; R: reverse primers used for PCR amplification.

### DNA purification

PCR products were gel purified in a 2% low melt agarose gel using QIAquick® Gel Extraction Kit (Qiagen Inc., Valencia, CA) per manufacturer's recommendations. The extracted PCR products were visualized for purity on a 2% agarose-TAE/EtBr gel.

### Restriction Fragment Length Polymorphism (RFLP) Analysis

Gene segment sequences were analyzed using *RestrictionMapper* (http://www.restrictionmapper.org/) in order to identify restriction enzymes that recognized the least number of cut sites per gene segment. Purified segment specific DNA was digested with the respective restriction enzymes (Table 2). The digestion reactions were in 10 µl volume with 10 units of the designated enzyme and incubated for 3 hrs following reaction conditions recommended by the manufacturers (NEB, Ipswich, MA or Fermentas Inc., Glen Burnie, MD). The DNA from the hy donor virus, wt virus and their respective reassortants was digested concurrently. The digestion reactions were visualized on a 2% agarose-TAE/EtBr gel.

### Statistical analysis

Statistical significance of gene constellation frequency was analyzed using a chi-square test. Number Cruncher Statistical System (NCSS, Kaysville, UT) was used for the analysis. A value of *p*<0.05 was considered statistically significant.
